# Metagenomics Meets Electrochemistry: Utilizing the Huge Catalytic Potential From the Uncultured Microbial Majority for Energy-Storage

**DOI:** 10.3389/fbioe.2020.00567

**Published:** 2020-06-04

**Authors:** Nicole Adam, Stefanie Schlicht, Yuchen Han, Mikhael Bechelany, Julien Bachmann, Mirjam Perner

**Affiliations:** ^1^Molecular Biology of Microbial Consortia, Biocenter Klein Flottbek, University of Hamburg, Hamburg, Germany; ^2^Department of Chemistry and Pharmacy, Friedrich-Alexander University of Erlangen-Nürnberg, Interdisciplinary Center for Nanostructured Films (IZNF), Erlangen, Germany; ^3^Institut Européen des Membranes, IEM – UMR 5635, ENSCM, CNRS, Univ Montpellier, Montpellier, France; ^4^Institute of Chemistry, Saint-Petersburg State University, Universitetskiy Prospekt, St. Petersburg, Russia

**Keywords:** metagenomics, electrochemical cells, hydrogen production, hydrogenase, polyacrilonitrile fiber electrodes, energy storage

## Abstract

Hydrogen can in the future serve as an advantageous carrier of renewable energy if its production via water electrolysis and utilization in fuel cells are realized with high energy efficiency and non-precious electrocatalysts. In an unprecedented novel combination of structured electrodes with hydrogen converting enzymes from the uncultured and thus largely inaccessible microbial majority (>99%) we address this challenge. The geometrically defined electrodes with large specific surface area allow for low overpotentials and high energy efficiencies to be achieved. Enzymatic hydrogen evolution electrocatalysts are used as alternatives to noble metals. The enzymes are harnessed from the environmental microbial DNA (metagenomes) of hydrothermal vents exhibiting dynamic hydrogen and oxygen concentrations and are recovered via a recently developed novel activity-based screening tool. The screen enables us to target currently unrecognized hydrogenase enzymes from metagenomes via direct expression in a surrogate host microorganism. This circumvents the need for cultivation of the source organisms, the primary bottleneck when harnessing enzymes from microbes. One hydrogen converting metagenome-derived enzyme exhibited high activity and unusually high stability when dispersed on a TiO_2_-coated polyacrylonitrile fiber electrode. Our results highlight the tremendous potential of enzymes derived from uncultured microorganisms for applications in energy conversion and storage technologies.

## Introduction

Humanity’s ever-growing energy demand, the finite nature of fossil fuels, and the resulting CO_2_ emissions have encouraged researchers to find cost-efficient ways for generating energy in high quantities from renewable sources. Common fossil fuel alternative sources with high energy output include wind and solar power, but since they only deliver energy intermittently, a major problem is associated with energy storage (Armaroli and Balzani, 2011; Wang and Wang, 2015; Notton et al., 2018). An ideal energy storage medium is hydrogen. It can be generated by water electrolysis and can thus be easily applied for energy production in fuel cells. Hydrogen’s reactivity and pollutant-free combustion with oxygen makes it a renewable, clean energy storage medium (Armaroli and Balzani, 2011; Rosen and Koohi-Fayegh, 2016; Staffell et al., 2019). So far, large-scale hydrogen applications have been hampered by its inefficient, energy-expensive production, its delicate storage, and by the costly fuel cell technologies relying on rare noble metals such as platinum (Pt) (Schlapbach and Zuttel, 2001; Armstrong et al., 2009; Chenevier et al., 2013; Staffell et al., 2019). Moreso, the commonly used hydrogen production based on steam reforming of fossil fuels leads to carbon monoxide and sulfide impurities. Even traces of these impurities cause technical issues as they poison Pt electrodes used in fuel cells (Garcia and Koper, 2011; Liu et al., 2013; Staffell et al., 2019).

Given these drawbacks, hydrogen converting enzymes (hydrogenases) have gained more and more attraction: either as biological models for novel artificial catalysts or for direct application in biofuel cells or hydrogen production (Armstrong et al., 2009; Chenevier et al., 2013; Mazurenko et al., 2017; Esmieu et al., 2018; Le Goff and Holzinger, 2018; Qiu et al., 2019). Hydrogenases catalyze the interconversion of molecular hydrogen to protons and electrons (H_2_ ↔ 2 H^+^ + 2 e^–^) (Vignais and Billoud, 2007; Greening et al., 2016). These fundamentally bidirectional enzymes usually favor one way of the reaction but overall the direction is controlled by the redox potentials of the reactants (Vignais and Colbeau, 2004; Schilter et al., 2016). Although hydrogenases have been successfully applied to electrodes in electrochemical cells – for hydrogen oxidation as well as hydrogen production -, hydrogenase availability has had to rely on the cultivability of hydrogen converting microorganisms (Karyakin et al., 2005; Vincent et al., 2005; Hambourger et al., 2008; Wait et al., 2010; Schlicht et al., 2016; Mazurenko et al., 2017; Szczesny et al., 2018). The tremendous hydrogenase resource amongst the uncultured majority of microbes (depending on the type of habitat up to 99% of strains; Lloyd et al., 2018) has not been accessible. However, this has changed with the advent of metagenomic tools which provide access to the total DNA of an environmental sample, including that of uncultured microorganisms (Handelsman et al., 1998).

We recently developed an activity-based screen for seeking hydrogen converting enzymes from the metagenome (Adam and Perner, 2017), circumventing the need for culturing the hydrogen converting organisms directly. Particularly, hydrothermal vents are promising habitats to screen for hydrogenases (Adam and Perner, 2018a), since hydrogen is released here from inner earth, providing an energy source for chemosynthetic microbes. Our recent screening activities of hydrothermal vent metagenomes recovered four hydrogen converting active clones. They mostly exhibited low or even no sequence similarity to known hydrogenases or other enzymes and demonstrated the large potential for hydrogen converting enzymes from uncultured microorganisms derived from hydrothermal vent environments (Adam and Perner, 2018b). Here, we report on the immobilization of one of these enzymes on electrode surfaces of large specific surface area for hydrogen production.

## Methods

### Recovery of Hydrogen Uptake Active Fosmid Clones

The detailed steps and procedures for recovering hydrogen converting fosmid clones have previously been published (Adam and Perner, 2017, 2018b). In short: A piece of a massive sulfide chimney from the Sisters Peak hydrothermal vent system, high-temperature fluids from the Nibelungen vent field and low-temperature fluids from the Lilliput venting site were collected from the southern Mid-Atlantic Ridge using the remotely operated vehicle (ROV) KIEL6000 (GEOMAR) during the MAR-SUED cruise in 2009. From each sample metagenomic DNA was extracted and amplified via multiple displacement amplification as described before (Han and Perner, 2014; Böhnke and Perner, 2015). Metagenomic libraries were constructed using the CopyControl^TM^ Fosmid Library Production Kit and the broad host range vector pRS44 (Aakvik et al., 2009; Adam and Perner, 2017). The metagenomic fosmids were transferred into a [NiFe]-hydrogenase deletion mutant of *Shewanella oneidensis* MR-1 (*S. oneidensis* Δ *hyaB*) via triparental mating and the resulting clones were screened for hydrogen uptake activity. The screen is based on the reduction of Fe(III)citrate (included in the medium for the chemolithotrophic growth of *S. oneidensis*) coupled to the oxidation of molecular hydrogen. This redox reaction results in a color change of the medium and allows the identification of hydrogen uptake active clones. The metagenomic inserts of hydrogen uptake active clones were sequenced and deposited at the National Center for Biotechnology Information (NCBI) under Genbank accession numbers MG456603-MG456606 (Adam and Perner, 2018b). One of the two hydrogen uptake active metagenomic clones identified in the Sisters Peak library was SP11F2 (Adam and Perner, 2018b), later chosen for the application on nanoporous fiber electrodes.

### Bacterial Strains, Growth Conditions, and Hydrogen Evolution Assay

*Shewanella oneidensis* Δ*hyaB* derived stains were routinely grown at 28°C in lysogeny broth (LB) medium. For hydrogenase assays, these strains were grown anaerobically in fresh water medium (FW medium, supplemented with 15 mM pyruvate and 15 mM fumarate) with H_2_/CO_2_ (80%/20%, 1 atm) (Westfalen AG, Münster, Germany) in the headspace (Lovley et al., 1989; Han and Perner, 2014; Adam and Perner, 2018b). If required, antibiotics were used at the following concentrations: kanamycin 20 μg mL^–1^, gentamycin 10 μg mL^–1^, and chloramphenicol 12.5 μg mL^–1^. *S. denitrificans* was routinely grown at 22°C in DSMZ medium 113 under anaerobic conditions with H_2_/CO_2_ (80%/20%, 1 atm) in the headspace (Han and Perner, 2014).

For the hydrogen evolution assays subcellular fractionations were performed in an anaerobic chamber (Coy Laboratory Products, Grass Lake, MI, United States) as previously described (Maroti et al., 2009; Han and Perner, 2014; Adam and Perner, 2018b). The hydrogen evolution assay was performed as described before (Nishihara et al., 1997; Maroti et al., 2009) by measuring the evolution of hydrogen from methyl viologen (MV), reduced by sodium dithionite (Merck KGaA, Darmstadt, Germany). Routinely, the reaction mixture containing 100 μL of 400 mM oxidized MV, 25 μg protein of the membrane fraction and 1.5 mL of 20 mM sodium phosphate buffer (pH 7.0, with 1 mM DTT) was sealed in a 15 mL Hungate tube and flushed with nitrogen gas (Westfalen AG, Münster, Germany) for 2 min. The reaction was initiated by adding 100 μL of 5% (w/v) sodium dithionite solution. After 1 h incubation at 28°C, the reaction was terminated by adding 500 μL of 40% (w/v) sodium trichloroacetate solution. One milliliter gas sample from the headspace was injected into a gas chromatograph (GC; Thermo Fischer Scientific Inc., Waltham, MA, United States) to determine the amount of evolved hydrogen as previously described (Han and Perner, 2014). For the determination of the pH optimum, the experiments were performed using 20 mM sodium phosphate buffer (containing 1 mM DTT) and the following pH values: 5.0, 6.0, 7.0, 8.0, and 9.0. Enzyme stability was tested by performing the hydrogen evolution assay at pH 7.0 with membrane fractions that were stored for 24 h: (i) anoxic (i.e., under an atmosphere of 97% N_2_ and 3% H_2_) at 4°C, (ii) oxic, (i.e., under ambient air) at 4°C, (iii) anoxic at room temperature (22°C), and (iv) oxic at room temperature. The specific hydrogen evolution activities were calculated from three independent measurements.

### Electrode Preparation

The procedures for electrode preparation have been modified from a previous publication (Schlicht et al., 2016). The polyacrylonitrile (PAN) fibers were synthesized by electrospinning and coated with a thin TiO_2_ layer (15 nm) by atomic layer deposition (ALD) in order to gain an electrically conductive layer. The ALD process was performed at 120°C in a GEMSTAR 6 reactor (Arradiance LLC, Sudbury, MA, United States) using titanium tetraisopropoxide (TTIP) and H_2_O. For the Ti precursor the pulse duration, exposure and pump time were set to 2, 30, and 60 s, respectively. The corresponding durations for water were set to 0.2, 30, and 60 s. The thickness of the TiO_2_ layer was determined on a Si wafer by spectroscopic ellipsometry using a SENpro (SENTECH Instruments GmbH, Berlin, Germany). Finally, the TiO_2_ layer was annealed at 400°C in air for 1 h in a P 330 muffle furnace (Nabertherm GmbH, Lilienthal, Germany).

### Electrochemical Studies

For the electrochemical performance measurements the coated fibers were cut into small pieces and glued with double-sided Cu tape onto Al plates. To define an accurate specific surface area a polyamide tape (Kapton©, DowDuPont, Midland, MI, United States) mask with a laser-cut circular opening of diameter *d* = 3 mm was used. Cyclic voltammetry (CV) and steady-state electrolysis were performed in a standard three-electrode setup with an Ag/AgCl reference electrode and a Pt mesh as the counter-electrode using a Gamry Interface 1000 potentiostat (Gamry Instruments, Warminster, PA, United States). The 20 mM NaH_2_PO_4_/Na_2_HPO_4_ buffer electrolyte solution was degassed with N_2_ bubbling for 30 min before each experiment. Each membrane fraction was either used in its pure form or at a 1:10 dilution. Before the hydrogenase extract was applied, all electrodes were investigated without any treatment by CV starting from –0.6 V between –0.4 and –1.0 V, followed by steady-state electrolysis at –1.0 V (η = –0.39 V) for 30 min. These data were used as the reference for subsequent electrocatalysis. Afterward, the electrodes were dried at 50*°*C for 1 h. A 10 μL droplet of the membrane fractions of either P5H2 or SP11F2 was then added and after a waiting time of 10 min to allow for the proteins to adhere to the fibers’ surface the same measurement procedure (CV and electrolysis) was performed. The measurements were repeated without any further sample treatment on later days.

## Results and Discussion

**FIGURE 1:**
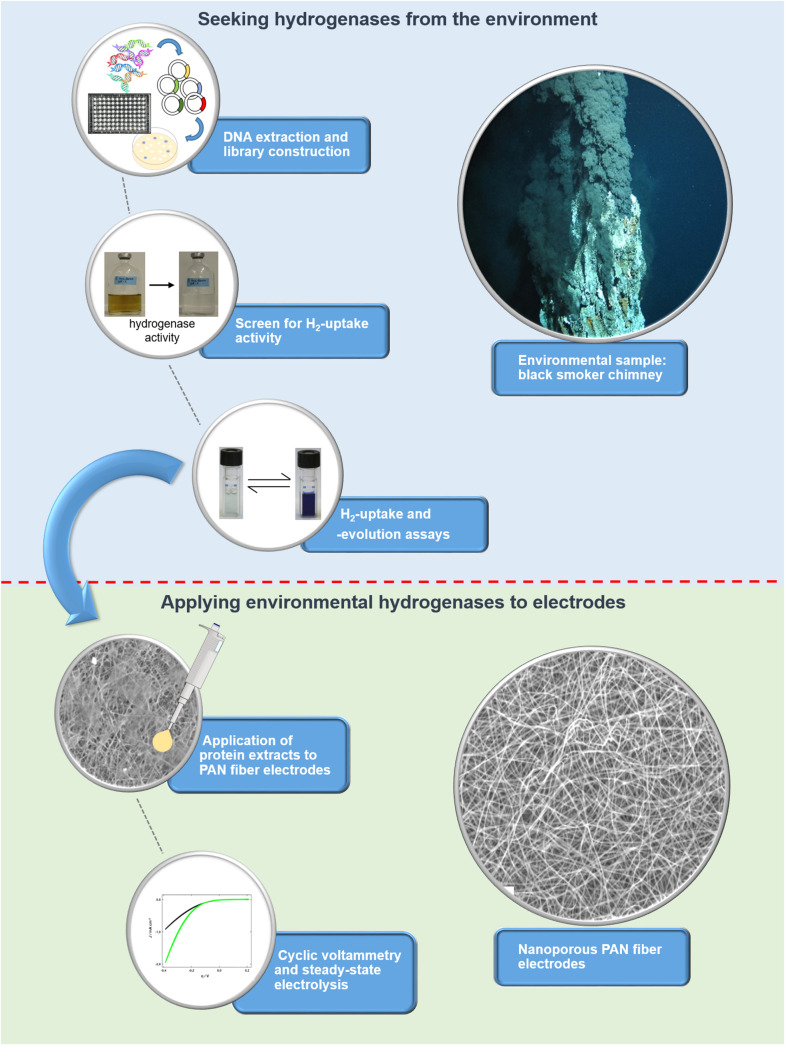
Overview of the workflow for identifying (blue background) and applying metagenomic hydrogen converting enzymes to electrodes with large specific surface area (green background). The screen for hydrogen uptake activity is based on a color change of the medium as a result of the reduction of Fe(III)citrate, which can only take place if hydrogen is oxidized by a hydrogen converting enzyme.

To unlock the hydrogen converting potential amongst uncultured microorganisms, we recently developed an activity-based screen for the identification of hydrogen uptake active clones from metagenomes and applied it to hydrothermal vent samples (Adam and Perner, 2017, 2018b). Briefly, the metagenomic DNA is captured in a broad-host range fosmid vector and is then expressed in a foreign host, in our case a *Shewanella oneidensis* MR-1 hydrogenase deletion mutant (Δ*hyaB*). *S. oneidensis* Δ*hyaB* lacks the ability to produce an active hydrogenase, but the hydrogen uptake (i.e., hydrogen oxidation) activity can be restored by complementing the mutant with an intact hydrogenase gene. It was shown that *S. oneidensis’* hydrogenase maturation apparatus (encoded by several accessory genes) can successfully assemble and fold hydrogenases from phylogenetically diverse Proteobacteria (Adam and Perner, 2018b). Thus, the screening procedure is suitable for the identification of various hydrogen uptake active enzymes from the environment. We screened 14,400 fosmid clones derived from metagenomic DNA of three different hydrothermal vent systems and identified four clones with hydrogen uptake and *in vivo* hydrogen consumption activity (Adam and Perner, 2018b). Except for one clone (Lilli33G1, harboring a *Wolinella succinogenes* hydrogenase homolog), no homologs to known hydrogenase-coding genes could be identified in the metagenomic DNA inserts. For the present study, the most active hydrogen-evolving clone (SP11F2) was chosen for further experiments, including stability tests (at different temperatures and oxygen levels) and testing the pH range of the recombinant, partially purified enzyme. Subsequently, it was immobilized on TiO_2_-coated polyacrylonitrile (PAN) fiber electrodes of large specific surface area to produce hydrogen and thereby convert electrical energy into chemical that is into storable fuel. For an overview of the individual steps from the discovery to the point of application of the metagenomic hydrogen converting enzyme (see Figure 1).

### Hydrogen Evolution Activities of Hydrogenases From Uncultured Bacteria in *S. oneidensis* Δ*hyaB*

**FIGURE 2:**
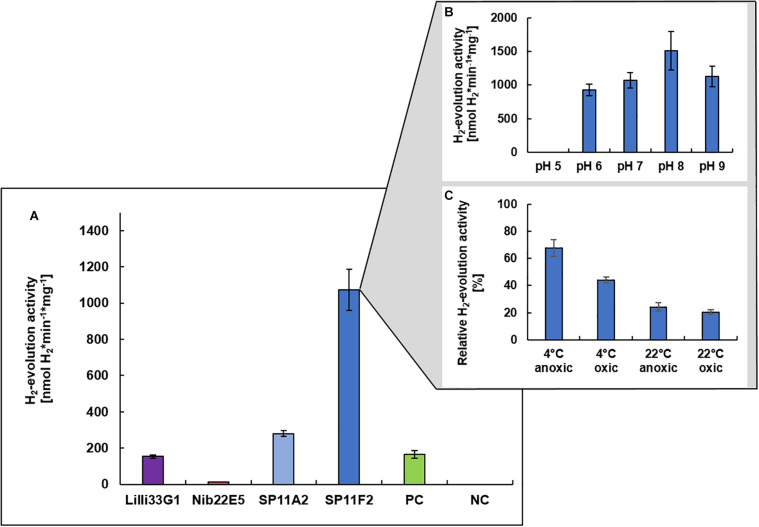
H_2_-evolution activities of recombinant hydrogen converting enzymes. Specific H_2_-evolution activities of the membrane fractions of four metagenomic clones (measured at pH 7.0) are shown **(A)**. The respective metagenomic DNA was derived from three different hydrothermal vent systems from the Mid-Atlantic Ridge: (i) the Lilliput system (Lilli33G1, marked in purple), (ii) the Nibelungen system (Nib22E5, marked in red), and (iii) the Sisters Peak vent (SP11A2 and SP11F2, marked in blue). The positive control (marked in green) is the membrane fraction of clone P5H2 (harboring the recombinant *S. oneidensis* wild type hydrogenase) and the negative control is the membrane fraction of *S. oneidensis* Δ*hyaB* harboring the empty pRS44 vector, which is used for the heterologous expression of (meta-) genomic inserts. The pH optimum of SP11F2 is shown in **(B)** and the stability of SP11F2 at pH 7 under different storage conditions (for 24 h) has been tested **(C)**. Anoxic conditions refer to an atmosphere of 97% N_2_ and 3% H_2_, oxic conditions to ambient air (21% O_2_) and the relative activities refer to the original measurement of 1073 nmol H_2_*min^–1^*mg^–1^, displayed in panel **(A)**. *n* = 3 for all samples.

Prior to the immobilization of a metagenomic hydrogen converting enzyme on the PAN fiber electrodes, the hydrogen evolution (i.e., hydrogen production) activities of the four discovered hydrogen uptake active clones (Nib22E5, SP11A2, SP11F2, and Lilli33G1) were determined under neutral pH conditions to identify the most promising candidate. Except for clone Nib22E5, the hydrogen evolution activities of the metagenomic clones were all higher than that of clone P5H2, which is the mutant *S. oneidensis* Δ*hyaB* complemented with *S. oneidensis*’ own hydrogenase gene (*S. oneidensis* Δ*hyaB:hyaB_*S. oneidensis*_*). SP11F2 had the highest hydrogen evolution activity rates: the partially purified membrane fraction of SP11F2 exhibited with 1073 ± 115 nmol H_2_^∗^min^–1*^mg^–1^ a more than 6-fold higher activity than that of P5H2 at 28°C (Figure 2A). The subsequent analysis of the pH optimum showed a peak at pH 8.0 with an even 41% higher activity than at pH 7.0 (Figure 2B). However, for the following application in electrochemical cells, the H_2_-evolution activity at pH 7.0 was the determining factor. The stability tests with SP11F2’s membrane fraction (stored for 24 h under different conditions) showed, that the activity of hydrogen evolving enzyme can (although not fully) be restored after exposure to atmospheric oxygen levels (Figure 2C). In these experiments, temperature appears to have the highest influence on the hydrogen evolution activity: while after storage at 4°C still 67% (under anoxic conditions) and 44% (oxic conditions) of the original activity at pH 7.0 could be observed, at room temperature, the activities dropped to 24% (anoxic conditions) and 20% (oxic conditions, Figure 2C). Intriguingly, in a previous study, SP11F2’s membrane fraction only showed hydrogen uptake activity at 55°C (175 ± 11 nmol H_2_^∗^min^–1*^mg^–1^ of partially purified protein) but no activity could be measured at 25°C. In summary, SP11F2 exhibits a very high hydrogen evolution activity at 28°C, while the only measured hydrogen uptake activity is relatively low (cf. Figure 2A and Adam and Perner, 2018b). This indicates that the respective hydrogenase performs better in the conditions of hydrogen evolution at 28°C. A classification of this hydrogen converting enzyme is not possible to date, given that no open reading frames (ORF) with homologies to known hydrogenases could be identified in the respective metagenomic DNA insert. The majority of the detected ORFs (Genbank accession number MG456604) was related to different hypothetical proteins of *Aquificales* with identities of up to 68% (Adam and Perner, 2018b).

The so far unknown hydrogen converting enzyme of clone SP11F2 also considerably exceeds the hydrogen evolution activities of some cultured representatives such as the *Gammaproteobacterium Hydrogenovibrio marinu*s (402 nmol H_2_^∗^min^–1*^mg^–1^ of partially purified protein; Nishihara et al., 1997) and the *Epsilonproteobacterium Sulfurimonas denitrificans* (248 ± 10 nmol H_2_^∗^min^–1*^mg^–1^ of partially purified protein, this study). Thus, SP11F2 displays the ideal candidate for putative applications in hydrogen production under pH neutral conditions and emphasizes the huge functional potential of so far unknown enzymes hidden among the uncultured majority of vent-associated microorganisms.

### Applying Hydrogen Converting Enzymes From an Uncultured Vent Organism to Nanoporous Surface-Enlarged Electrodes

**FIGURE 3:**
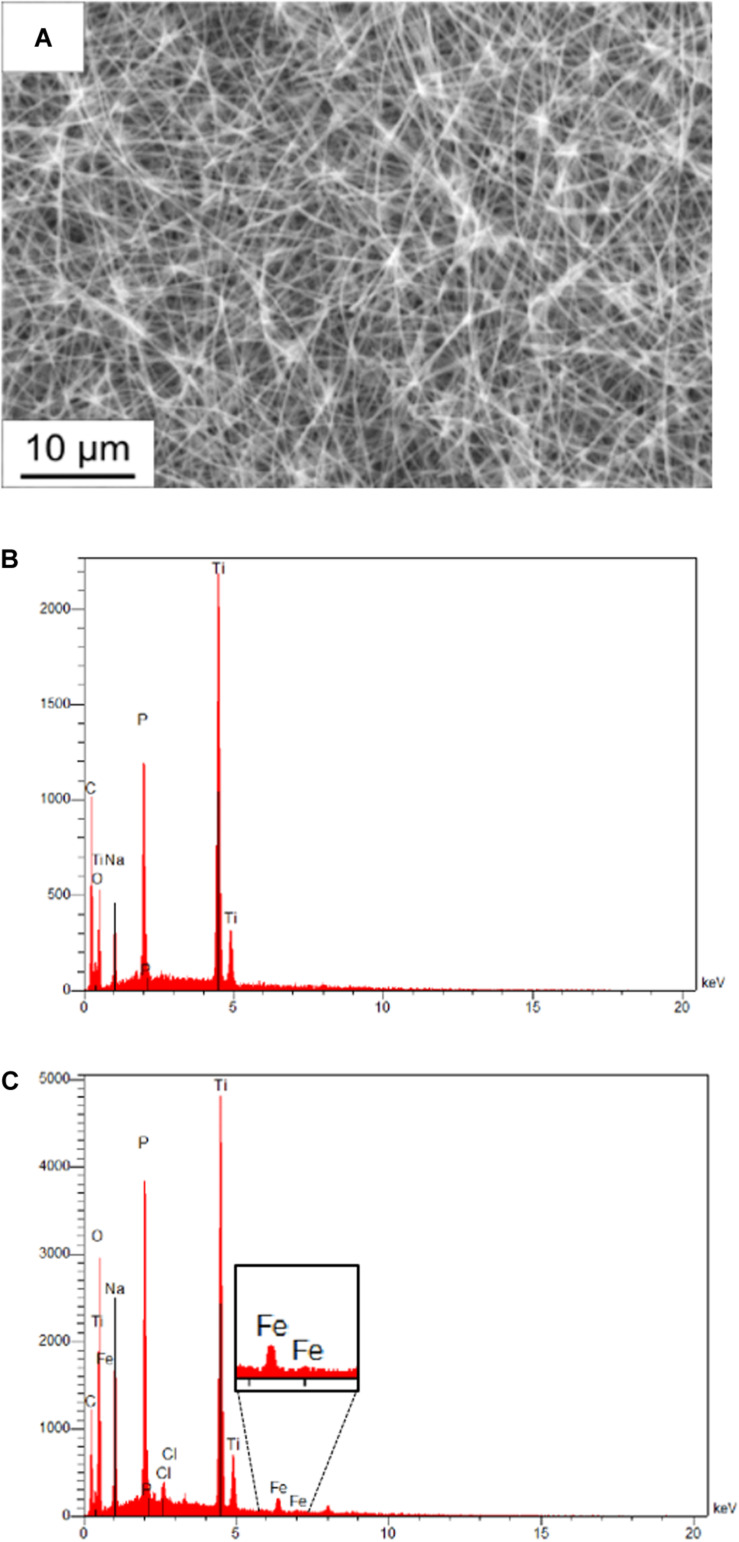
Scanning electron micrograph and energy-dispersive X-ray analysis of PAN fiber electrodes. **(A)** Scanning electron micrograph of a coated electrospun fiber after adding diluted hydrogenase fraction. The individual fibers of the mat are still visible, which maintains the activity of the whole surface area. **(B)** Energy dispersive X-ray analysis of a fiber mat without treatment and **(C)** of a fiber mat treated with pure membrane fraction after electrochemical investigation. The presence of the Fe signal proves the adhesion of hydrogenase-containing membrane fractions on the coated fibers.

The membrane fractions of the metagenomic clone SP11F2 and the complemented mutant P5H2 were applied to TiO_2_-coated PAN fiber electrodes with tunable, large specific surface areas. An electrospinning time of 6 h resulting in a large active surface area was selected, which remained after treatment of the electrodes with partially purified proteins. Energy-dispersive X-ray analysis confirmed the presence of the elements C, Ti, O of the coated fibers and Na and P from the electrolyte. The Fe signal after the electrochemical investigation verified the adhesion of the hydrogenase-containing membrane fraction on the fiber mat, also visible on the scanning electron micrograph (Figure 3).

**FIGURE 4:**
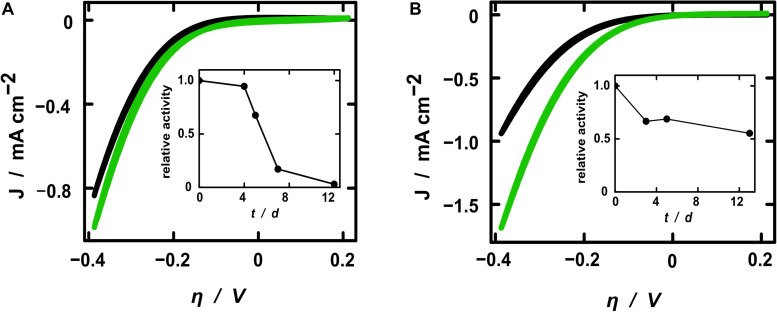
Cyclic voltammograms of electrospun fibers coated with TiO_2_. Current densities are shown for electrodes without (black curves) and with diluted H_2_-evolution active membrane fractions (green) of clone P5H2 [**(A)**, harboring the *S. oneidensis* wild type hydrogenase] and SP11F2 [**(B)**, harboring an unknown hydrogen converting enzyme]. Note that always the 20th CV cycle (reaching a stable state) is shown. Additionally, the stability of the H_2_-evolving enzymes is displayed as the relative activities of electrodes coated with the diluted membrane fractions of P5H2 **(A)** and SP11F2 **(B)**. At each time point the same electrochemical investigations (Cyclic voltammetry and electrolysis) were performed without any further treatment.

The electrochemical activity toward hydrogen evolution of the membrane fractions was investigated by cyclic voltammetry (CV) and steady-state electrolyses. Starting from –0.6 V, the cyclic voltammograms presented the expected exponential shape of the current density resulting from the hydrogen evolution reaction (Figure 4). All samples exhibited higher current densities after the addition of active protein extracts, whereby the pure fraction showed higher activity toward hydrogen evolution. This demonstrates that the increase of current density after treatment with the hydrogenase fraction resulted solely from the hydrogen converting enzymes itself and not from some other component of the electrolytic solution. The relative current enhancement after the addition of diluted SP11F2 membrane extracts was about 3-fold higher than with P5H2 (Figure 4), thereby demonstrating a less pronounced difference of the two membrane extracts compared to the respective specific hydrogen evolution activities (>6-fold higher activity of SP11F2, Figure 2). When undiluted membrane fractions were applied, no significant difference between the current densities of P5H2 and SP11F2 was visible (data not shown). This effect may be caused by the organic cell-derived compounds contained in the membrane fractions. These can cause a clogging of the PAN fibers and thus hinder the ion transport to and from the electrode surface.

In a previous study a [NiFe]-hydrogenase containing membrane fraction of *E. coli* was applied to PAN fiber electrodes in an analogous setup. Although the actual increase of the current densities does not depend on the enzymes alone, the metagenomic SP11F2 *S. oneidensis* Δ*hyaB* clone clearly exceeded the performance of the *E. coli* hydrogenase by more than 50% if applied to electrodes of comparable geometries (cf. Schlicht et al., 2016). To put the numbers in a broader perspective, the absolute current density values recorded here are certainly not comparable with those obtained in industrial electrolyzers of either the PEM (acidic) or alkaline type. However, even noble metal catalysts only reach current densities on the same order of magnitude as reported here if used in our mild, pH-neutral conditions (Hwang et al., 2015). The inherent safety of a pH 7 operation is crucial to decentralized, small-scale applications for individual consumer markets and thus represents another advantage of the hydrogenase-based system compared to the hitherto applied industrial electrolyzers that are typically operated at pH 0 or 14.

All electrochemical measurements were repeated on the following days without any further treatment in order to gain insight into the stability of the enzyme-coated PAN fiber electrodes. As expected, the relative activity decreased over time. However, the stability of the hydrogenase from the metagenomic clone SP11F2 exceeded that of clone P5H2 considerably: while the relative activity of the P5H2 electrode tended to zero after 12 days, the SP11F2 membrane extract coated on the electrode still retained more than 50% of its original activity after 12 days and storage at room temperature and an atmosphere containing 1% O_2_ (Figure 4). A comparison of these results with stability tests based on the H_2_-evolution assays (of the pure membrane extracts) suggests that the immobilization of the enzyme on the electrodes has a stabilizing effect. If not immobilized on PAN fiber electrodes, the enzyme-activity showed noticeable decreases already after 24 h storage at ambient temperature (under anoxic as well as fully oxic conditions). Under these conditions, only 24 and 20% of the original activity could be observed (Figure 2C). The storage at 4°C lead to higher activities after 24 h than the storage at room temperature (67 and 44%, respectively; Figure 2C). Still, it ranges far beyond the stability of the immobilized membrane fractions (cf. Figures 2C, 4). Although it does not reach the stability of *Thiocapsa roseopersicina* (half-life time of 60 days at 24°C) (Zorin and Gogotov, 1982) or *Alteromonas macleodii* (100% enzyme activity after 45 days at 4°C) (Vargas et al., 2011), SP11F2 exhibits a higher oxygen-tolerance and stability than the [NiFe]-hydrogenase of *S. oneidensis* (and the oxygen-sensitive majority of hydrogen converting enzymes; Vignais and Billoud, 2007), if immobilized on PAN fiber electrodes.

## Conclusion

Overall, our findings show the remarkable potential of “currently unknown” hydrogen converting enzymes for biotechnological applications. We found an enzyme that is: (i) able to oxidize or produce hydrogen (the latter at even higher rates than the hydrogenase of the host in which it is expressed), (ii) functions when immobilized on nanoporous electrodes, and (iii) exhibits a remarkable stability at ambient temperature and under the presence of low oxygen levels. Due to the inconceivably large pool of environmental hydrogen converting enzymes that still need to be explored, numerous hydrogenases exhibiting similar or even more extraordinary qualities are likely awaiting their discovery.

For future large-scale applications, these enzymes should ideally combine high hydrogen evolution activities, exceptional O_2_ tolerance and stability at ambient (or elevated) temperatures over long periods of time. A way to recover such an enzyme (in addition to searching for it with an activity-based screen) could be the optimization of highly active (metagenomic) hydrogenases such as that of clone SP11F2: for example, the O_2_ tolerance of a [NiFe]-hydrogenase could successfully be increased by targeted mutations of the hydrogen access channel (Dementin et al., 2009). Still, this would be very time-consuming for hydrogen converting enzymes with structural differences compared to the classical hydrogenases such as the here presented metagenomic hydrogen converting enzyme. Likely, in the same (or shorter) time a naturally O_2_-tolerant metagenomic hydrogenase could be found by means of our activity-based screen. Given the elevated hydrogen concentrations and steep thermal and chemical gradients dominating at deep-sea hydrothermal vent systems (Kelley et al., 2002; Han and Perner, 2014), the ideal enzyme combining high hydrogen turnover rates and stability is likely to be present in these habitats but has not been found yet. Since we have so far only investigated a tiny fraction (14,400 fosmid clones with metagenomic inserts) of the tremendous potential of hydrogen converting enzymes, we might one day be able to recover a hydrogenase suited for use on large electrodes in industrial-scale electrolyzers.

## Data Availability Statement

The datasets generated for this study are available on request to the corresponding author.

## Author Contributions

MP, MB, and JB planned the project. NA and MP wrote the manuscript with major contributions from JB and approval of all authors. NA and YH performed hydrogenase evolution assays and prepared membrane fractions for electrode coating. SS performed ALD, electrode preparation, and electrochemical investigation. MB was responsible for electrode design, mainly electrospinning.

## Conflict of Interest

The authors declare that the research was conducted in the absence of any commercial or financial relationships that could be construed as a potential conflict of interest.
